# Supplementary material for: Describing synchronization and topological excitations in arrays of magnetic spin torque oscillators through the Kuramoto model
Source: Sci Rep. 2016 Sep 1;6:32528. doi: 10.1038/srep32528 (PMC5007507; doi:10.1038/srep32528)
Supplement: Supplementary Information [file srep32528-s1.pdf]

Supplementary information: Describing synchronization and topological excitations in arrays of magnetic spin torque oscillators through the Kuramoto model

Vegard Flovik,[1,*] Ferran Macià,[2] and Erik Wahlström[1]

[1]*Department of Physics, NTNU, Norwegian University of Science and Technology, N-7491 Trondheim, Norway*

[2]*Institut de Ciència de Materials de Barcelona (ICMAB-CSIC), Campus UAB, 08193 Bellaterra, Spain*

From the Thiele equation to the phase oscillator model

We here provide some more details on the derivation of the Kuramoto model starting from the coupled Thiele equation:

$$G(\mathbf{e_z} \times \dot{\mathbf{X}}_{1,2}) - k(\mathbf{X}_{1,2})\mathbf{X}_{1,2} - D_{1,2}\dot{\mathbf{X}}_{1,2} - \mathbf{F}_{\text{STT}1,2} - \mathbf{F}_{\text{int}}(\mathbf{X}_{2,1}) = 0. \tag{S1}$$

Here, $G = -2\pi p M_s h/\gamma$ is the gyroconstant, p is the core polarity, γ is the gyromagnetic ratio, M_s is the saturation magnetization and h is the thickness of the ferromagnetic layer. The confining force is given by $k(\mathbf{X}_{1,2}) = \omega_{01,2}G\left(1 + a\frac{\mathbf{X}_{1,2}^2}{R_{1,2}}\right)$ [1, 2], where $R_{1,2}$ are the disc radii and the gyrotropic frequency for disc $1,2$ is $\omega_{01,2} = \frac{20}{9}\gamma M_s h/R_{1,2}$. The damping coeficcient $-D_{1,2} = \alpha\eta_{1,2}G$, where $\eta_{1,2} = \frac{1}{2}\ln\left(\frac{R_{1,2}}{2l_e}\right) + \frac{3}{8}$. Here, $l_e = \sqrt{\frac{A}{2\pi M_s}}$ is the exchange length given by the exchange stiffness A and the saturation magnetization M_s. Assuming a uniform perpendicularly magnetized polarizer layer, $\mathbf{F}_{\text{STT}} = \pi\gamma a_J M_s h(\mathbf{X}_{1,2} \times \mathbf{e_z}) = \varkappa(\mathbf{X}_{1,2} \times \mathbf{e_z})$ [3], where the spin torque coefficient is given by $a_J = \hbar p_z J/(2|e|hM_s)$, $\hbar$ is the Planck‘s constant, J is the current density and e is the elementary charge. The interaction between the neighboring vortices is summarized by a dipolar coupling term given by $\mathbf{F}_{\text{int}} = -\mu(d)\mathbf{X}_{2,1}$, where $\mu(d)$ describes the interaction strength as a function of the separation d between the STO. A study of the dipolar interaction between neighboring vortices has been performed by Araujo *et al.* [4]. Starting from a macrodipole approximation for the dipolar energy between two magnetic dipoles μ_1 and μ_2, they show that the average interaction energy can be written as $\langle W_{int}\rangle = \mu_{eff}C_1C_2X_1X_2$. Here, C_i and X_i are the chirality and gyration radius respectively and μ_{eff} is given by:

$$\mu_{\text{eff}} = 3\frac{\pi^2\chi^2R^2h^2}{2d^3}, \tag{S2}$$

where $\chi = 2/3$, R is the disc radius, h the thickness and d is the inter-disc spacing. In polar coordinates $(X_{1,2}\cos\theta_{1,2}, X_{1,2}\sin\theta_{1,2})$, the coupled equations for two neighboring vortices from Eq. (S1) can be written as:

$$\frac{\dot{X}_1}{X_1} = \alpha\eta_1\dot{\theta}_1 - \frac{\varkappa}{G} + \frac{\mu X_2}{GX_1}\sin(\theta_1 - \theta_2) \tag{S3}$$

$$\dot{\theta}_1 = -\frac{k(X_1)}{G} - \alpha\eta_1\frac{\dot{X}_1}{X_1} - \frac{\mu X_2}{GX_1}\cos(\theta_1 - \theta_2) \tag{S4}$$

$$\frac{\dot{X}_2}{X_2} = \alpha\eta_2\dot{\theta}_2 - \frac{\varkappa}{G} - \frac{\mu X_1}{GX_2}\sin(\theta_1 - \theta_2) \tag{S5}$$

$$\dot{\theta}_2 = -\frac{k(X_2)}{G} - \alpha\eta_2\frac{\dot{X}_2}{X_2} - \frac{\mu X_1}{GX_2}\cos(\theta_1 - \theta_2) \tag{S6}$$

One can then show that after a few approximations, the set of equations reduce to that of two coupled phase oscillators. We assume the same gyration radius for both vortices, $X_2 = X_1$, and that the steady state vortex gyrotropic radius is close to its mean value, X_0. This means that Eq. (S3) can be set to zero, as $\dot{X}_1 = 0$, and we obtain:

$$\dot{\theta}_1 = \frac{\varkappa}{\alpha\eta_1 G} - \frac{\mu}{\alpha\eta_1 G}\sin(\theta_1 - \theta_2) \tag{S7}$$

Setting $\dot{X}_1 = 0$ and $X_2 = X_1$ also in Eq. (S4):

$$\dot{\theta}_1 = -\frac{k(X_1)}{G} - \frac{\mu}{G}\cos(\theta_1 - \theta_2) \tag{S8}$$

We then add Eqs. (S7) and (S8) to obtain:

$$\dot{\theta}_1 = \frac{\varkappa - \alpha\eta_1 k(X_1)}{2\alpha\eta_1 G} - \frac{\mu}{2\alpha\eta_1 G}\left[\sin(\theta_1 - \theta_2) + \alpha\eta_1\cos(\theta_1 - \theta_2)\right]. \tag{S9}$$

Following the same procedure for vortex nr. 2 and assuming low damping, $\alpha\eta << 1$, we obtain the equations for two coupled phase oscillators θ_1 and θ_2:

$$\dot{\theta}_1 = \omega_1 + \lambda\sin(\theta_2 - \theta_1), \tag{S10}$$

$$\dot{\theta}_2 = \omega_2 + \lambda\sin(\theta_1 - \theta_2), \tag{S11}$$

Where $\omega_{1,2} = \frac{\varkappa - \alpha\eta_{1,2}k(X_{1,2})}{2\alpha\eta_{1,2}G}$ and $\lambda = \frac{\mu}{2\alpha\eta_{1,2}G}$. The functional form of Eqs. (S10)-(S11) is the same as that of the well known Kuramoto model, which is a generalization for the case of an ensemble of weakly coupled phase oscillators. Considering the interaction between several STO, determined by the interaction strength λ_{ij} between oscillators θ_i and θ_j, we obtain a

Kuramoto model for a population of N interacting oscillators:

$$\frac{d\theta_i}{dt} = \omega_i + \sum_{j\neq i} \lambda_{ij} \sin(\theta_j - \theta_i). \tag{S12}$$

Vortex annihilation processes

Starting from a disordered initial condition, a number of vortices with $n = \pm 1$ is created initially, depending on the array size. Thermal fluctuations of sufficient amplitude could give rise to vortex unbinding, where free vortices proliferate due to thermal fluctuations. As we do not consider thermal effects, such vortex unbinding is not observed this in our model. Since a vortex is topological, it exists until it meets and annihilates with a vortex of opposite polarity, and the transition from disordered to a synchronized state is governed by vortex annihilation processes.

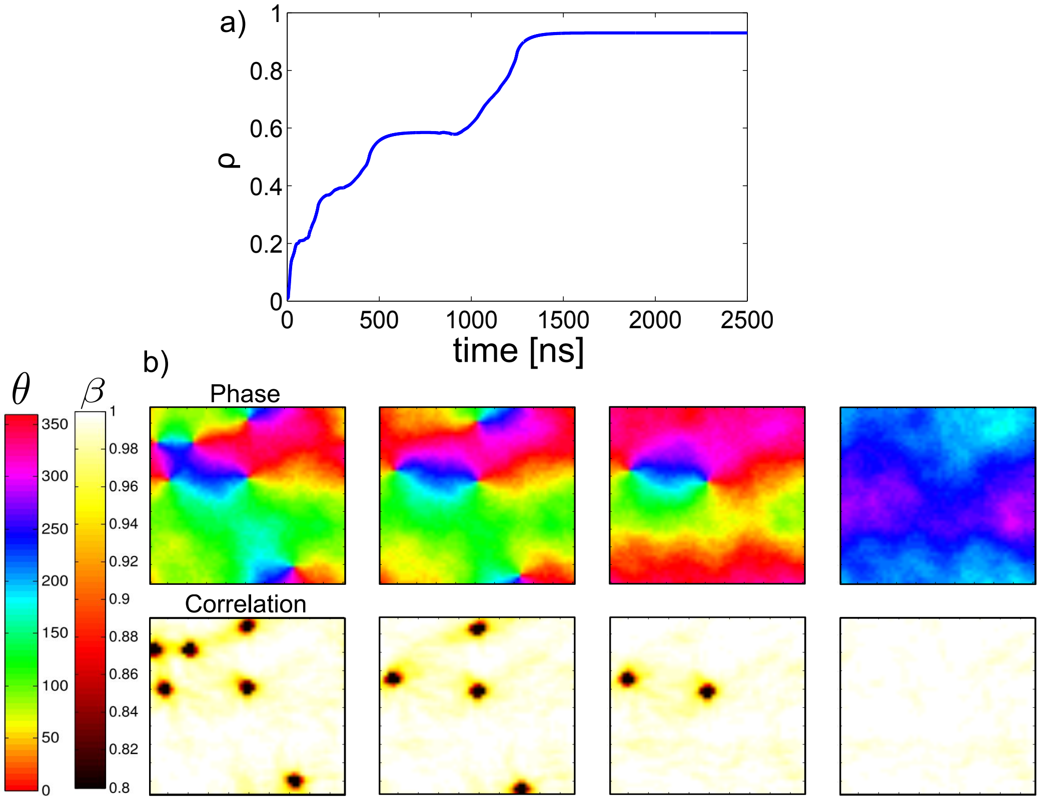

FIG. S1. a) Order parameter ρ vs. time for an interaction strength of $\lambda = 25$ MHz for a system of 50×50 oscillators, starting from a disordered initial state. b) Snapshots of phase and correlation maps at various timesteps (increasing time from left to right), showing the vortex annihilation processes.

In Fig. S1a we show the order parameter ρ vs. time, starting from a disordered initial state for a system of 50×50 oscillators using the Kuramoto model. The observed jumps in the order parameter correspond to the annihilation of vortices of charge ± 1. This process is also illustrated in the panels of Fig. S1b, where we show snapshots of the phase map θ_i and local correlation β_i at various timesteps (with time increasing from left to right). The location and polarity ($n = \pm 1$) of the vortices can be seen in the phase maps in the upper panels. The position of the vortex core is identified by areas of low correlation ($\beta \to 0$) between neighboring oscillators, seen as the black spots in the lower panels. As time progress the vortices annihilate, resulting in a globally synchronized and phase coherent state.

Correlation function and correlation length

The spatial correlation function is given asymptotically by: $\langle\theta(\mathrm{r})\cdot\theta(\mathrm{R})\rangle \propto e^{-|r-R|/\xi}/|r-R|^{\eta}$. The brackets indicate the correlation between oscillators at positions r and R, and the correlation length ξ is obtained by averaging over all positions r and R in the array. An example of the decay of spatial correlations is shown in Fig. S2 for a system of 50×50 oscillators using the Kuramoto model, showing a dominating exponential decay in the correlations for increasing distances between the oscillators. The spacing $|r - R|$ is here expressed in terms of the number of lattice spacings between the oscillators. From the decay of the correlation function, we can then extract the correlation length ξ.

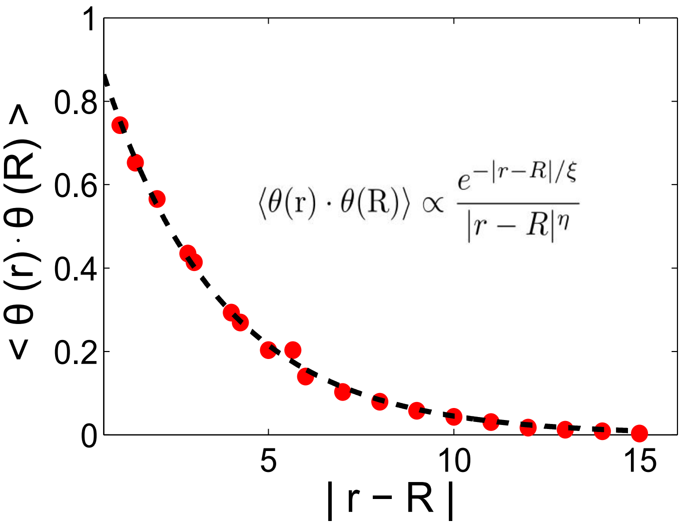

FIG. S2. Correlation as a function of oscillator spacing, $|r - R|$ for a system of 50×50 oscillators using the Kuramoto model.

* vflovik@gmail.com

[1] Guslienko, K. Yu., Han, X. F., Keavney, D. J., Divan, R., Bader, S. D. Magnetic Vortex Core Dynamics in Cylindrical Ferromagnetic Dots. Phys. Rev. Lett. 96, 067205 (2006).

[2] Ivanov, B. A., Zaspel, C. E. Excitation of Spin Dynamics by Spin-Polarized Current in Vortex State Magnetic Disks. Phys. Rev. Lett. 99, 247208, (2007).

[3] Khvalkovskiy, A. V., Grollier, J., Dussaux, A., Zvezdin, A., Cros, V. Vortex oscillations induced by spin-polarized current in a magnetic nanopillar: Analytical versus micromagnetic calculations. Phys. Rev. B 80, 140401(R) (2009).

[4] Araujo, F. A. *et. al.* Optimizing magnetodipolar interactions for synchronizing vortex based spin-torque nano-oscillators. Phys. Rev. B 92, 045419 (2015).
